# LC-MS/MS Determination and Pharmacokinetic Study of Dehydrocorydaline in Rat Plasma after Oral Administration of Dehydrocorydaline and *Corydalis yanhusuo* Extract

**DOI:** 10.3390/molecules191016312

**Published:** 2014-10-13

**Authors:** Qiu-Yue Li, Kai-Tong Li, Hong Sun, Wen Jin, Jia-Wen Shi, Yue Shi

**Affiliations:** Institute of Medicinal Plant Development, Chinese Academy of Medical Sciences & Peking Union Medical College, No. 151, Malianwa North Road, Haidian District, Beijing 100193, China; E-Mails: liqiuyue@implad.ac.cn (Q.-Y.L.); likaitong@joinn-lab.com (K.-T.L); hsun@implad.ac.cn (H.S.); wjin@implad.ac.cn (W.J.); jiawen86712@163.com (J.-W.S.)

**Keywords:** LC-MS/MS, dehydrocorydaline, pharmacokinetics, *Corydalis yanhusuo*

## Abstract

A rapid, sensitive and selective liquid chromatography/tandem mass spectrometry method (LC-MS/MS) was developed and validated for determination of dehydrocorydaline (DHC) in rat plasma using nitidine chloride as an internal standard. The analytes were solid-phase extracted and eluted on a C18 chromatography column using a mobile phase of acetonitrile and water (containing 0.8% formic acid and 10 mM ammonium acetate) (28:72, v/v). Detection was performed using positive ion electrospray ionization in multiple reaction monitoring modes. The assay was linear over the concentration range 0.625–250 ng/mL with a quantification limit of 0.625 ng/mL. The precision was <13.7%, the accuracy >93.1%, and extraction recovery ranged from 92.1% to 107%. The validated method was successfully applied to the pharmacokinetics and excretion study of DHC in rat plasma after oral administration of pure DHC and an effective fraction of *Corydalis yanhusuo* (EFY). The pharmacokinetic parameters showed that DHC from EFY was absorbed more rapidly and eliminated more slowly than pure DHC. The result suggests that the differences might be due to the presence of P-glycoprotein (P-gp) inhibitors and that other alkaloids co-existing in the EFY may compete with DHC for transportation by P-gp, metabolization by P450, and binding to plasma proteins.

## 1. Introduction

**Figure 1:**
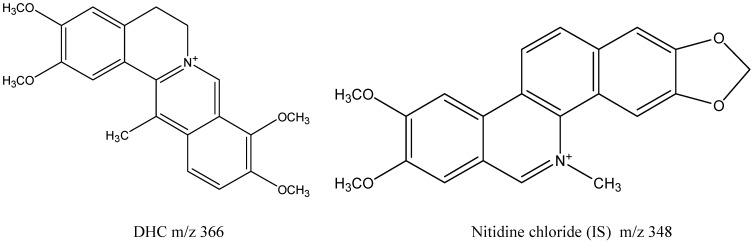
Chemical structures of dehydrocorydaline (DHC) and nitidine chloride (IS).

Dehydrocorydaline (DHC), a quaternary protoberberine-type alkaloid (Figure 1), is an important bioactive component of a very well-known Chinese herbal medicine, Yan-Hu-Suo, which is the tuber of *Corydalis yanhusuo*. The herb has been widely used as an analgesic agent for treating spastic, abdominal, and menstrual pains, and for pain due to injury [1]. It has also been widely used to promote blood circulation and treat coronary heart disease [2,3,4,5]. Previous studies have identified that quaternary and tertiary alkaloids are the active secondary metabolites of the plant [6,7]. The pharmacological effects of the two different types of alkaloid focus on different aspects. Tertiary alkaloids have been found to be effective at alleviating pain (e.g., tetrahydropalmatine, corydaline, and protopine, in order of decreasing efficiency [1]). In contrast, some quaternary alkaloids, such as DHC, have been found to be more active than tertiary alkaloids in increasing the tolerance of mice to monobasic and hypobaric hypoxia. They also have greater efficacy than tetrahydropalmatine (a tertiary alkaloid) in protecting cardiomyocytes against ischemia in the myocardium *in vitro*. As well as two other quaternary alkaloids, berberine and palmatine, it appears that these are the main active ingredients in *Corydalis yanhusuo* for the treatment of coronary heart disease [8,9,10].

**Figure 2:**
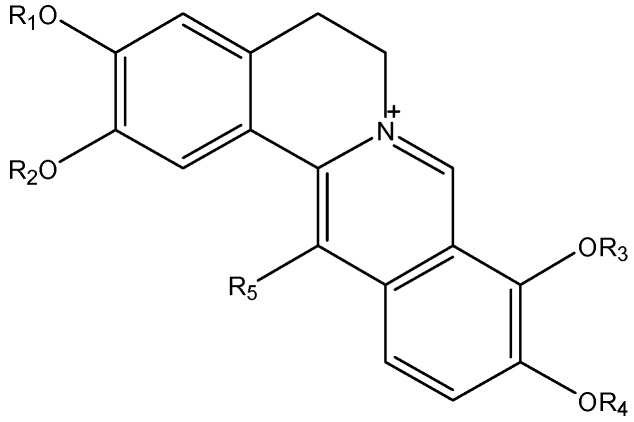
The quaternary alkaloids from EFY.

Through qualitative and quantitative research on the effective fraction of *Corydalis yanhusuo* (EFY) [11,12,13,14,15,16], we have found that quaternary alkaloids are the main bioactive components (Figure 2). In addition, our Caco-2 cell experiment [17] suggested that synergy between the alkaloids in the fraction might promote their oral absorption across the intestinal epithelium. On the other hand, another study showed that the plasma concentration–time curve for DHC can only be obtained when it is given orally in high doses after a total alkaloid extraction from *Rhizoma Corydalis*, indicating that DHC is absorbed poorly [18]. There are, at present, large differences in the studies on the absorption of quaternary protoberberine-type alkaloids.

**Table:** 

Quaternary Alkaloids	R1	R2	R3	R4	R5
13-methyl-palmatrubine	CH_3_	CH_3_	H	CH_3_	CH_3_
13-methyl-dehydrocorydalmine	CH_3_	CH_3_	CH_3_	H	CH_3_
dehydrocorydaline	CH_3_	CH_3_	CH_3_	CH_3_	CH_3_
dehydrocorybulbine	CH_3_	H	CH_3_	CH_3_	CH_3_
columbamine	CH_3_	H	CH_3_	CH_3_	H
coptisine	-CH_2_-		-CH_2_-		H
berberine	-CH_2_-		CH_3_	CH_3_	H
palmatine	CH_3_	CH_3_	CH_3_	CH_3_	H

On the one hand, quaternary protoberberine-type alkaloids are considered to be poorly absorbed because, possibly, of the action of P-glycoprotein (P-gp) inhibitors resulting in trace plasma concentrations after oral administration. On the other hand, it was found that the absorbed amounts of berberine, a quaternary protoberberine-type alkaloid, accounted for 33.6% of the given dose 1 h after oral administration in an *in situ* assay of the intestinal loop [19]. Berberine may be quickly excreted from the blood in rats through bile [20,21]. This indicates that DHC can be absorbed well but has a low dosage in systemic circulation. This might be because there may be a very large “first pass” effect in the liver after oral administration. Therefore, data on the cumulative excretion of unchanged quaternary protoberberine-type alkaloids in the bile after oral administration would be very important for clarifying the absorption extent.

Up to now, only two papers investigated the pharmacokinetics of DHC [18,22]. In this paper, a highly sensitive liquid chromatography–tandem mass spectrometry (LC-MS/MS) method is developed for DHC determination. Comparing with the published reports, our method with isocratic elution in R.T. (retention time) is much better, the LLOQ is 0.625 ng/mL. With SPE, we got high recovery and less matrix effect. It is subsequently successfully applied to a pharmacokinetic study of DHC when given to rats in two different dosage forms: one, an effective fraction of *Corydalis yanhusuo*, and the other, the pure compound. After oral administration, the results were analyzed to see if there were any synergies between the compounds in EFY and to further assess whether the influence of other compounds in EFY affected the pharmacokinetics and blood concentration of DHC. In addition, excretion of DHC in the bile and urine of the rats was investigated to clarify the pharmacokinetic properties of DHC.

## 2. Results and Discussion

### 2.1. Method Validation of DHC in Rat Plasma

**Figure 3:**
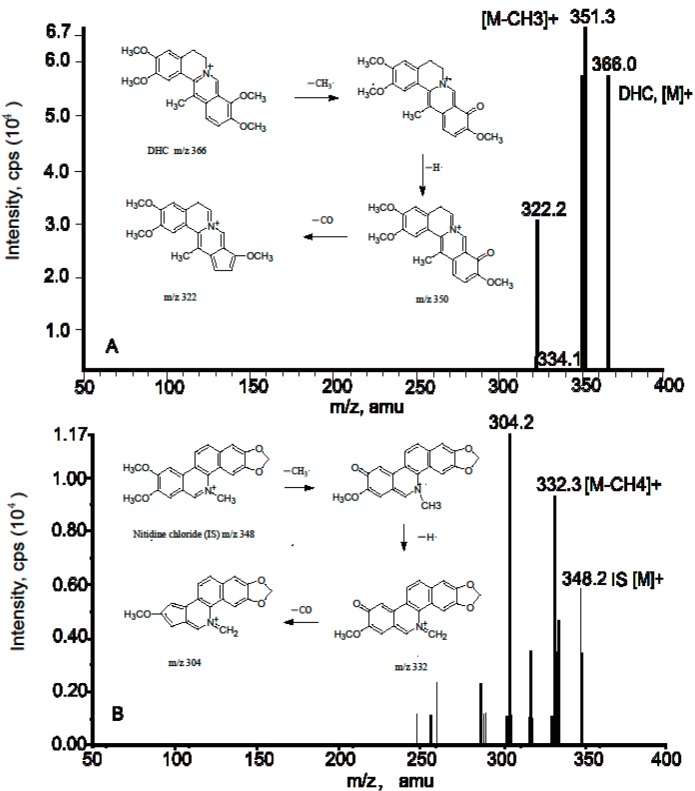
Positive-ion electrospray mass spectra for DHC (**A**) and IS (Internal Standard) (**B**).

**Figure 4:**
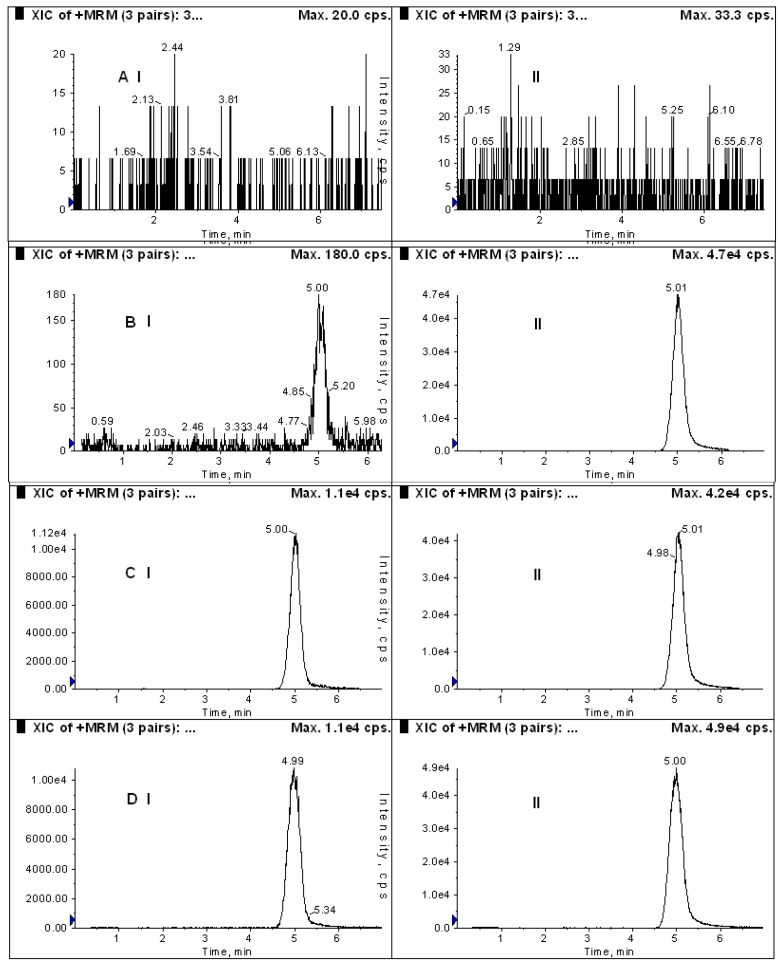
MRM chromatograms for (**A**) blank plasma, (**B**) plasma (200 μL) spiked with 2.5 and 800 ng/mL IS; (**C**) plasma spiked with 100, and 800 ng/mL IS; and (**D**) a plasma sample 1 h after oral administration of 97.5 mg/kg DHC to a rat. I: DHC; II: IS.

In order to investigate the comparative pharmacokinetics of DHC in rats, a sensitive and reliable analytical method was developed and validated. Assay selectivity was evaluated by analyzing blank plasma samples from six rats. Positive-ion electrospray mass spectra for DHC and IS are shown in Figure 3. The peaks [M]^+^ = 366 and 348 for DHC and IS, respectively, were chosen for quantification purposes due to their high stability and intensity. Figure 4 shows representative MRM chromatograms of blank plasma (A), plasma spiked with 2.5 ng/mL DHC and 800 ng/mL IS (B), plasma spiked with 100 ng/mL DHC and 800 ng/mL IS (C), and a plasma sample 1 h after oral administration of 97.5 mg/kg DHC to a rat. No interfering peaks were observed in the blank plasma under assay conditions.

A calibration curve was constructed using unweighted linear regression of the DHC/IS peak-area ratio (*Y*) against the corresponding spiked plasma DHC concentration in ng/mL (*X*) over the range 0.625–250 ng/mL. The regression equation had the form *Y* = 0.00916*X* + 0.000813 (r = 0.9976, n = 8). The concentrations in unknown samples were subsequently calculated using this calibration expression. The LLOQ of the assay was 0.625 ng/mL for DHC. The LLOQ, as the lowest concentration, was determined with both accurately and precisely. The precision (RSD, %) was 8.45 and the accuracy (RE, %) was 6.24. The results showed LLOQ of DHC can achieve 0.625 ng/mL for established method (Table 1).

**Table 1:** Accuracy and precision results for analysis of DHC in rat plasma (3 days, six replicates per day).

C_nom_ (ng/mL)	C_det_ (ng/mL)	Precision (RSD%)		Accuracy (RE%)
		Intra-day	Inter-day	
0.625	0.664	8.45	-	6.24
1.25	1.2	3.29	13.68	−6.89
25	23.7	4.06	7.07	−5.29
250	258.1	3.27	7.39	3.22

The precision and accuracy data for the assays is shown in Table 1. The results indicate that the LC-MS/MS method developed has good reproducibility with precision less than 13.68% and excellent accuracy ranging from −6.89% at low (1.25 ng/mL) to 3.22% at high (250 ng/mL) concentrations. Extraction recovery of the DHC was found to be satisfactory with average values ranging from 92.08% to 107.00% at the three QC concentrations (Table 2).

**Table 2:** The extraction recovery results for DHC in rat plasma (n = 6).

Concentration (ng/mL)	Recovery (%) (mean ± SD)	
	DHC	IS
1.25	107 ± 2.31	
25	92.08 ± 3.82	74.56 ± 4.05
250	99.65 ± 4.07	

The stability study showed that the variation in the concentration after three cycles of freezing and thawing was within ±15% of the nominal concentration, indicating no significant loss of substance during freezing and thawing. When the processed samples were stored in an auto-sampler at 4 °C, the DHC also showed good stability. This is evidenced by the fact that the responses varied no more than ±10% over 24 h at the QC concentrations. After storage at ambient temperature for 12 h, the concentration of DHC in the plasma deviated less than ±13% from those in freshly-spiked plasma (Table 3).

**Table 3:** Stability results for analysis of DHC in rat plasma (n = 6).

Storage	C_nom_ (ng/mL)	C_dec_ (ng/mL) (mean ± S.D.)	Relative Error (%)	RSD (%)
**Postpreparative stability (24 h)**	1.25	1.18 ± 0.06	−5.47	5.26
	25	26.2 ± 0.86	4.8	8.13
	250	270.33 ± 9.42	8.14	3.48
**Stability after three freeze-thaw cycles**	1.25	1.25 ± 0.1	−0.27	9.86
	25	27.5 ± 0.87	10	3.17
	250	272.67 ± 8.14	9.07	2.99
**Short-term temperature stability (12 h)**	1.25	1.2 ± 0.1	−3.87	8.14
	25	21.97 ± 0.51	−12.13	2.33
	250	230.67 ± 15.96	−7.73	6.92

As shown in Table 4, no significant matrix effect was observed for the six blank plasma lots, indicating that the extracts were “clean” with no co-eluting of “unseen” compounds that could influence the ionization of DHC.

**Table 4:** Matrix effects data for DHC at 25.0 ng/mL and IS 200 ng/mL in six different sources of rat plasma.

	Matrix Effect (%) (mean ± S.D.)	RSD (%)
DHC	89.40 ± 0.03	3.1
IS	92.3 ± 0.04	4.1

### 2.2. Method Validation of DHC in Bile and Urine Samples

For biliary and urinary excretion study, the calibration curves were constructed using unweighted linear regression of the DHC/IS peak-area ratio (*Y*) against the corresponding spiked plasma DHC concentration in ng/mL (*X*), the ranges are 1.25–500 ng/mL for biliary and 0.625–250 ng/mL for urinary. The regression equations had the form *Y* = 0.000437*X* + 0.0149 (r = 0.9971, n = 8) and *Y* = 0.00675*X* + 0.00279 (r = 0.9984, n = 8). The concentrations in unknown samples were subsequently calculated using this calibration expression. The LLOQ of the assay was 1.25 ng/mL in bile and 0.625 ng/mL in urine. Assay selectivity was evaluated by analyzing blank samples from four rats. Results showed that no interfering peaks were observed in both blank bile and urine under assay conditions.

### 2.3. Application

#### 2.3.1. Plasma Pharmacokinetics

**Figure 5:**
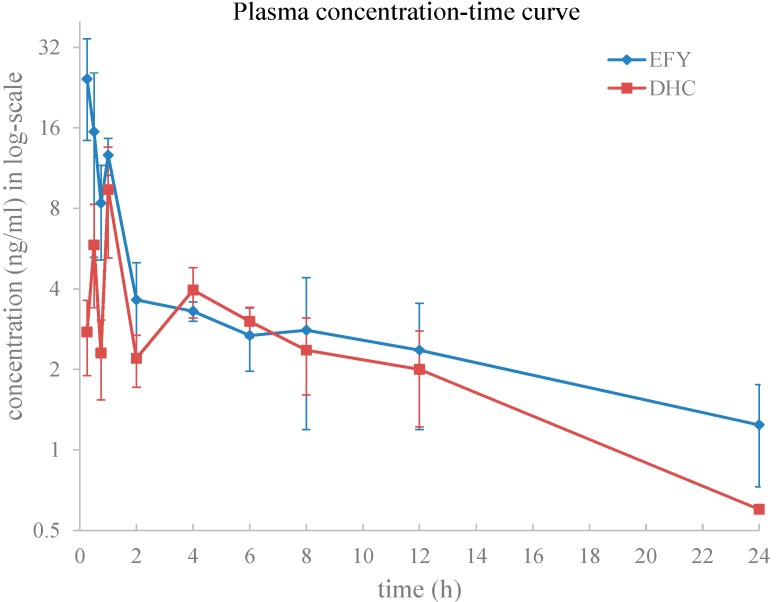
Mean plasma concentration in log-scale-time profiles of DHC in rats following oral administration either in an EFY form or in pure form at an equivalent dose of 97.5 mg/kg.

DHC is the most abundant quaternary protoberberine-type alkaloid in EFY and has clear cardiovascular pharmacological activity. A fully validated LC-ESI-MS/MS method was developed and was successfully applied to a pharmacokinetic study of DHC when given in the two different dosage forms (EFY and pure compound) via oral administration in rats. The doses of DHC and EFY were 97.5 mg/kg and 483 mg/kg (equivalent to 97.5 mg/kg DHC), respectively. The mean plasma concentration-time profiles of DHC after the two doses are illustrated in Figure 5 and the estimated pharmacokinetic parameters are presented in Table 5.

Except for the AUC_0–*t*_ parameter of DHC, all the other pharmacokinetic parameters were statistically significantly different (*p* < 0.05) between the two dosage forms. The *C*_max_ and AUC_0–∞_ of DHC after being given in EFY form were about 3.1- and 1.9-fold higher than those when given as the pure compound, respectively. The *t*_1/2_ of the DHC was also changed, being about 2.7-fold longer in the EFY form than in the pure form. The *T*_max_ of the DHC was decreased 0.31-fold shorter than when given as pure compound. These results indicate that, when given in mixture form (*i.e.*, EFY), DHC seems to be absorbed faster and eliminated more slowly from the body compared to administration of the pure substance.

**Table 5:** Pharamcokinetic parameters of DHC in rats following oral administration, either in an EFY form or in pure form, at an equivalent dose of 97.5 mg/kg.

Parameter	Dosage Forms		*p* Value
	EFY	DHC	
C_max_ (ng/mL)	28.7 ± 6.92	9.40 ± 4.18	0.001
T_max_ (h)	0.31 ± 0.13	1.00 ± 0.00	0.000
AUC_0-t_ (ng∙h/mL)	71.92 ± 14.79	52.39 ± 12.82	0.056
AUC_0-∞_ (ng∙h/mL)	115.12 ± 34.12	59.08 ± 11.53	0.008
T_1/2_ (h)	21.71 ± 12.35	7.93 ± 1.34	0.038
K_e_ (1/h)	0.05 ± 0.03	0.09 ± 0.02	0.023

The differences in the pharmacokinetic parameters of DHC after oral administration of pure DHC and EFY demonstrate that other components co-existing in the EFY appear to affect the absorption and elimination of DHC. However, the mechanisms for this interaction are not yet clear. Nevertheless, there are two basic conclusions that are clear from earlier studies.

First, berberine is a substrate of P-glycoprotein since P-gp is involved in the efflux activity of berberine in Caco-2 cells and the liver [23,24]—it has an obscure function to increase or inhibit P-gp function. Although there are not any reports that DHC is a substrate of P-gp, DHC has structure similar to berberine, it is also presumably a substrate of P-pg. Thus, we can infer that other alkaloids in EFY, which have structures similar to berberine’s, are most likely to be substrates of P-gp. They will compete with DHC for the P-gp involved in the efflux activity and so indirectly decrease the amount of DHC effluxed by P-gp. This will increase the DHC blood concentration and prolong elimination time.

Secondly, the process of drug combining with plasma protein is usually reversible. When the process is inhibited by combined drug and other reasons, the concentration of free drugs will greatly increase. Therefore, the mutual competition of several drugs will lead to drug concentration and other parameters changing rapidly while they are sharing limited binding site. It seems from former studies that berberine might be a weak cytochrome P450 3A4 inhibitor [25]. As a result, it is reasonable to reach the inference that other alkaloids in EFY with similar structures to berberine are likely to enter into the competition for biding sites with DHC for enzymes involved in metabolism in the liver (and other organs). Therefore, the metabolic rate of DHC is decreased and the half-life of DHC becomes longer as a result. Up to now, there are no reports about binding values of DHC. *In vivo* metabolism of DHC was investigated, and several metabolites of DHC were detected and characterized by LC-MS. The research results will be published elsewhere.

Overall, compared with rats given pure DHC, the reason for the significant changes occurring in DHC’s pharmacokinetic parameters after oral administration of EFY may be that other components co-existing in EFY inhibit P-gp function. The other components co-existing in EFY may compete with DHC for P-gp transportation, metabolization by P450, and binding to plasma proteins. The mechanisms for these interactions should be investigated further.

#### 2.3.2. Excretion

**Figure 6:**
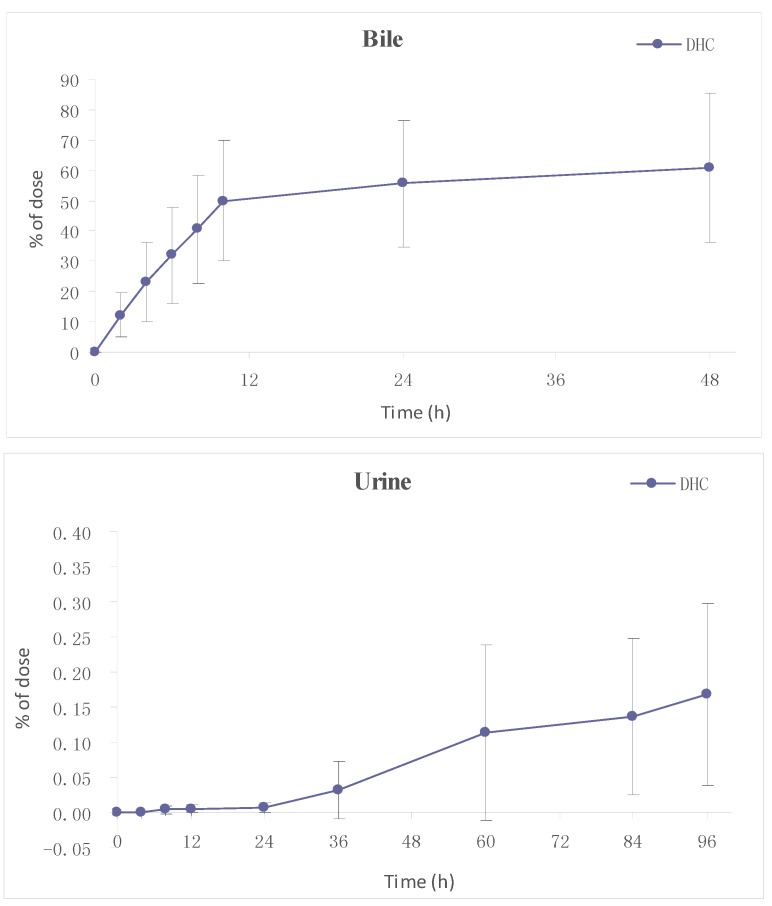
Cumulative amounts of DHC excreted in bile and urine after an oral dose of 97.5 mg/kg to rats.

The cumulative excretion of DHC in bile and urine after a single i.g. dose of DHC (97.5 mg/kg) was determined. Results (Figure 6) showed that there are wide individual differences in the biliary cumulative excretion of unchanged DHC. After a single oral dose of DHC, the rate of total biliary excretion was 60.04% ± 24.66% of the dose up to 48 h. However, the urinary cumulative excretion of DHC was little. The mean accumulated amount of DHC in urine over 96 h was 0.17% ± 0.13% of dose. Above results showed that DHC was mainly excreted in bile after oral administration of DHC. The mean accumulated amount of DHC in bile in just 2 h was 12.2% ± 7.38% of dose, indicating that DHC can be absorbed quickly following oral administration. There might be a huge “first pass” effect in liver after oral administration and account for the trace plasma concentration.

## 3. Experimental Section

### 3.1. Chemicals and Reagents

The nitidine chloride used as internal standard (IS) was purchased from the National Institute for the Control of Pharmaceutical and Biological Products (Beijing, China). DHC was isolated from the dried tuber of *Corydalis yanhusuo*. Its purity was found to be 99.6% using HPLC with photodiode array detection. The effective fraction of *Corydalis yanhusuo* (containing DHC 20.2%) was prepared in our laboratory. The methanol and acetonitrile used were of HPLC grade and were obtained from Fisher Co. Ltd. (Emerson, IA, USA). The formic acid and other reagents used were of analytical grade and purchased from Beijing Chemical Reagent Company (Beijing, China). Milli-Q water (Milford, MA, USA) was used throughout the study.

### 3.2. Animals and Doses

Specific pathogen free male Sprague-Dawley rats (weight: 220 ± 20 g) were obtained from the Institute of Laboratory Animal Science, Chinese Academy of Medical Sciences, Beijing, China. They were kept for 7 days in a controlled environment (22–24 °C, 60% relative humidity) under a 12 h light-dark cycle with free access to a soy-free custom diet and tap water. All the rats were fasted for 12–16 h before the experiment. All animal procedures were performed in compliance with the guidelines approved by the Animal Ethnics Committee of the Chinese Academy of Medical Sciences.

For pharmacokinetics study, the rats were divided into two groups (n = 5 per group). Doses of 97.5 mg/kg of DHC or 483 mg/kg of EFY (equivalent to 97.5 mg/kg DHC, 20.19% DHC in EFY) were all gavage administrated. Blood samples (500 μL) were withdrawn from the oculi chorioideae vein into heparinized tubes before dosing and subsequently at 15, 30 and 45 min and then at 1, 2, 4, 6, 8, 12 and 24 h following initial administration. After 1 h and 8 h, 2 mL of normal saline was injected into the caudal vein to compensate for blood loss. After centrifuging at 4000 rpm for 10 min, plasma samples were obtained and frozen at −20 °C until analysis.

For biliary excretion study, four rats were light anesthetized by ether and a PE-10 cannula was implanted into the bile duct to collect bile. A single dose of DHC was administered as described above. Bile samples were collected at 0–2, 2−4, 4−6, 6−8, 8−10, 10−24 and 24−48 h post-dosing and kept at −20 °C after the volume of each collection was recorded.

For urinary excretion study, four rats were used. DHC was administered as described above. The animals were housed in stainless steel metabolic cages with free access to tap water. Urine was collected at 0–4, 4–8, 8–12, 12–24, 24–36, 36–60, 60–84 and 84–96 h post-dosing and kept at −20 °C after the volume of each collection was recorded.

### 3.3. Preparation of Calibration Standards and Quality Control Samples

Stock solutions of DHC and IS were both prepared in methanol at a final concentration of 1 mg/mL and stored at −80 °C. A series of working standard solutions of DHC ranging from 2.5 to 1000 ng/mL and an IS solution at 800 ng/mL were prepared by diluting their stock solutions with 10% acetonitrile (stored for less than one week at −20 °C for the assay). Calibration standards were prepared using blank rat plasma (200 μL) spiked with 50 μL of DHC working solutions, to yield solutions with concentrations of 0.625, 1.25, 2.5, 6.25, 25, 62.5, 125 and 250 ng/mL. Quality control (QC) samples were prepared in the same way as the calibration samples at 1.25, 25.0 and 250.0 ng/mL, representing low, medium, and high concentrations of DHC in the plasma, respectively. The linearity for DHC in bile and urine established separately to be within 1.25–500 ng/mL and 0.625–250 ng/mL range.

### 3.4. Sample Preparation

A 200 μL aliquot of plasma was vortex mixed for 3 min with 200 μL of water and 50 μL of IS (800 ng/mL). After centrifugation at 9000 rpm for 10 min, all the supernatant was loaded onto an Oasis HLB cartridge (Waters, Milford, MA, USA), which was pre-conditioned with 1 mL of methanol followed by 1 mL of deionized water. The loaded-cartridge was sequentially washed with 1 mL of water and 1 mL of 20% methanol. The analytes were then eluted with 1 mL of methanol in water containing 2% formic acid. The collected eluate was evaporated to dryness at 40 °C under a gentle stream of nitrogen. The residue was reconstituted in 150 μL of 10% acetonitrile, and 30 μL of the resulting sample was injected into the LC-MS/MS system for assay. Bile and urine samples were all processed in a similar manner as the plasma samples.

### 3.5. LC-MS/MS Condition

An Agilent 1100 system (Palo Alto, CA, USA) equipped with a vacuum degasser, a quaternary pump, and an auto-sampler were used. Chromatographic separation was achieved on an Atlantis T3 column (100 mm × 2.1 mm, 5 μm; Waters, Milford, MA, USA), which was eluted with a mobile phase of acetonitrile and water (containing 0.8% formic acid and 10 mM ammonium acetate) (28:72, v/v). The mobile phase was delivered at a flow-rate of 0.25 mL/min and 40% of the eluate was introduced into an API 3000 triple-quadrupole mass spectrometer (Applied Biosystems MDS SCIEX, Concord, ON, Canada) equipped with a TurboIonSpray electrospray ionization (ESI) source. Detection was performed using positive-ion electrospray ionization in multiple reaction monitoring (MRM) modes. The transitions from molecular ion to dominant product ion *m/z* 366→351[M−CH_3_] and *m/z* 348→332[M−CH_3_−H] [26,27,28] were monitored for DHC and IS, respectively. The optimized working parameters for mass detection were as follows. The nebulizer, curtain, and collision-activated dissociation gases were set at 11, 7 and 11 (instrument units), respectively. The TurboIonSpray voltage and temperature were set at 4.5 kV and 400 °C, respectively. All data acquisition was performed using Analyst software (v1.4, AB MDS SCIEX).

### 3.6. Method Validation

#### 3.6.1. Calibration Linearity and Low Quantification Limits

A calibration curve for DHC was constructed by plotting the DHC/IS peak-area ratios *vs.* concentration of DHC in the plasma. Linearity was determined using linear least-squares regression. The lower limit of quantification (LLOQ) of the assay was defined as the lowest concentration on the standard curve that can be quantified with accuracy within ±15% bias of the nominal concentration and precision not exceeding 15% CV for DHC.

#### 3.6.2. Precision, Accuracy and Extraction Recovery

The precision (relative standard deviation) and accuracy (%) of the assay were determined from replicate analyses (n = 6). Three QC analyses were made on the same day (“intra-day”) and the others on 3 consecutive days (“inter-day”). The accuracy was calculated from the nominal concentration (*C*_nom_) and the mean value of the observed concentration (*C*_obs_) as follows:

bias (%) = [(*C*_obs_ − *C*_nom_)/*C*_nom_] × 100 (1)

The extraction recovery for DHC and IS was measured by comparing the peak areas of the extracted (pre-spiked) QC samples with those of the unextracted biological samples at an equivalent concentration. The recovery of DHC was determined at three levels (1.25, 25 and 250 ng/mL), while IS at a single concentration of 200 ng/mL. The results were expressed as mean ± standard deviation (SD).

#### 3.6.3. Stability

The stability of DHC in rat plasma was evaluated under conditions that mimicked those likely to be encountered during sample storage and analytical processing. Six replicates of QC samples were analyzed for DHC. The QC samples were frozen and stored at −20 °C for a week. The concentration variation of the DHC in the prepared QC samples was detected after (i) three cycles of freezing and thawing, (ii) placement in an auto-sampler at 4 °C for 24 h, and (iii) storing at ambient temperature for 12 h.

#### 3.6.4. Matrix Effects

Matrix effects (co-eluting undetected endogenous matrix compounds that may influence ionization of the DHC) were examined by comparing the DHC and IS peak-areas between two different sets of samples. In Set 1, standard DHC was dissolved in the reconstitution solvent and analyzed at DHC concentrations of 1.25, 25 and 250 ng/mL and an IS concentration of 200 ng/mL. These analyses were repeated six times at each concentration. In Set 2, blank plasma samples obtained from six rats were extracted and then spiked with the same concentration of DHC and IS dissolved in the reconstitution solvent. Deviation between the mean peak-areas of Set 2 *vs.* Set 1 indicates the possibility of ionization suppression or enhancement for DHC and IS. This is called an “absolute” matrix effect [29].

### 3.7. Pharmacokinetic Study

Pharmacokinetic parameters were established from the plasma concentration–time data using non-compartmental analysis. The terminal elimination rate constant (*K_e_*) was determined by linear regression of the terminal portion of the plasma concentration-time data. The elimination half-life (*t*_1/2_) was calculated using the expression:

*t*_1/2_ = ln2/*K_e_* (2)

The area under the curve (AUC) for the plasma concentration-time trace from zero to the last plasma drug concentration (AUC_0–*t*_) was calculated using the trapezoidal rule. Extrapolation to infinite time (AUC_0–∞_) was calculated using the expression:

AUC_0−∞_ = AUC_0−*t*_ + *C_t_*/*K_e_* (3)

where *C_t_* is the last measurable plasma concentration. The value of the maximal plasma concentration (*C*_max_) and time to maximal concentration (*T*_max_) were obtained directly from the plasma concentration-time curve.

The statistical significance of the pharmacokinetic parameters obtained from the two different forms (mixture/pure) was estimated using analysis of variance (ANOVA). SPSS 10.0 one-way ANOVA tests were used to compare the pharmacokinetic parameters from DHC and the mixture. A *p* value of less than 0.05 was considered to be significantly different. All results were expressed as arithmetic mean ± standard deviation.

## 4. Conclusions

For the first time, a sensitive and reliable LC-MS/MS method has been developed for the determination of DHC in rat plasma using solid-phase extraction as sample clean-up procedure. Full validation indicated that the established method was excellent sensitivity, linearity, precision, and accuracy. The method was successfully applied to the pharmacokinetic study of DHC in rat plasma after oral administration of pure DHC and EFY. Significant differences of *T*max and *C*max showed that DHC from EFY reached the peak concentrations more rapidly with higher concentrations, which indicated that the influence of the herb-herb interactions on DHC and other components co-existing in the EFY should be considered. Although the mechanism was still ambiguous and complex, the knowledge obtained in this study might help to explain the pharmacological mechanism and evaluate the impact of the differences in the efficacy and safety in clinical applications of EFY. Further research is in progress.

## References

[1] Tang F.Y., Nie A.G. (2006). Overview of studies on Corydalis yanhusuo. J. Clin. Exp. Med..

[2] Ou J.J., Kong L., Pan C.S., Su X.Y., Lei X.Y., Zou H.F.J. (2006). Determination of DL-tetrahydropalmatine in Corydalis yanhusuo by L-tetrahydropalmatine imprinted monolithic column coupling with reversed-phase high performance liquid chromatography. J. Chromatogr. A.

[3] Ling H.Y., Wu L.M., Li L.D. (2006). Corydalis yanhusuo rhizoma extract reduces infarct size and improves heart function during myocardial ischemia/reperfusion by inhibiting apoptosis in rats. Phytother. Res..

[4] Wu L.M., Ling H.Y., Li L.D., Jiang L.X., He M.L. (2007). Beneficial effects of the extract from Corydalis yanhusuo in rats with heart failure following myocardial infarction. J. Pharm. Pharmacol..

[5] Wen C.P., Wu L.M., Ling H.Y., Li L.D. (2007). Salutary effects of Corydalis yanhusuo extract on cardiac hypertrophy due to pressure overload in rats. J. Pharm. Pharmacol..

[6] Zhang J., Jin Y., Dong J., Xiao Y.S., Feng J.T., Xue X.Y., Zhang X.L., Liang X.M. (2009). Systematic screening and characterization of tertiary and quaternary alkaloids from *corydalis yanhusuo* W.T. Wang using ultra-performance liquid chromatography-quadrupole-time-of-flight mass spectrometry. Talanta.

[7] Yang X.B., Yang X.W., Liu J.X. (2014). Study on material base of Corydalis Rhizoma. China J. Chin. Mater. Med..

[8] Qin Y.Q., Yang Q.Z. (1978). Studies on chemical constituents for the treatment of coronary heart disease (CHD) effective components of Corydalis yanhusuo. Tianjin Med. J..

[9] Jiang X.R., Wu Q.X., Shi H.L., Chen W.P., Chang S.Q., Zhao S.Y., Tian X.Y., Zhou L.F., Guo S.M., Li Y.J. (1982). Pharmacological actions of dehydrocorydaline cardiovascular system. Acta Pharm. Sin..

[10] Li P., Ren J.G., Duan C.L., Lin C.R., Liu J.X. (2010). Effects of four components of Rhizoma Corydalis on anoxia and peroxidation injuries in neonatal cardiomyocytes. Chin. J. Chin. Mater. Med..

[11] Cheng X.Y., Shi Y., Zheng S.L., Jin W., Sun H. (2008). Two new protoberberine quaternary alkaloids from Corydalis yanhusuo. J. Asian Nat. Prod. Res..

[12] Cheng X.Y., Shi Y., Zheng S.L., Sun H., Jin W. (2008). Studies on chemical constituents in the anti-myocardial ischemia effective fraction of Corydalis yanhusuo. Chin. Med. Mat..

[13] Cheng X.Y., Shi Y. (2008). Analysis of chemical constituents of anti-myocardial ischemia fraction of *Corydalis* yanhusuo. Chin. J. Chin. Mater. Med..

[14] Cheng X.Y., Shi Y., Sun H., Jin W., Zheng S.L., Li K.T., Yang S. (2009). Identification and analysis of absorbed components in rat plasma after oral administration of active fraction of *Corydalis* yanhusuo by LC-MS/MS. Acta Pharm. Sin..

[15] Cheng X.Y., Shi Y., Zheng S.L., Sun H., Jin W. (2010). HPLC-MS Analysis of the Ethanol Extract of *Corydalis* yanhusuo and Simultaneous Determination of Eight Protoberberine Quaternary Alkaloids in it by HPLC-DAD. J. Chromatogr. Sci..

[16] Li K.T., Xing D.M., Jin W., Sun H., Shi Y. (2011). Chemical fingerprint and metabolic fingerprint analysis of the medical extract of Corydalis yanhusuo by HPLC-UV and HPLC-MS methods. Asian J. Chem..

[17] Li Q.Y., Cheng X.Y., Wang J.F., Yan L.L., Shi Y. (2013). Absorption and transportation of quaternary alkaloids in Corydalis yanhusuo across Caco-2 cell monolayer model. Chin. Hosp. Pharm. J..

[18] Lin L., Liu J.X., Zhang Y., Lin C.R., Duan C.L. (2008). Pharmacokinetic studies of tetrahydropalmatine and dehydrocorydaline in rat after oral administration of Yanhusuo extraction by LC-MS /MS method. Acta Pharm. Sin..

[19] Zuo F., Nakamura N., Akao T., Hattori M. (2006). Pharmacokinetics of berberine and its main metabolites in conventional and pseudo germ-free rats determined by liquid chromatography/Ion trap mass spectrometry. Drug Metab. Dispos..

[20] Tsai P.L., Tsai T.H. (2004). Hepatobiliary excretion of berberine. Drug Metab. Dispos..

[21] Li H.L., Zhang W.D., Zhang C., Liu R.H., Wang X.W., Wang X.L., Zhu J.B., Chen C.L. (2006). Bioavailabilty and pharmacokinetics of four active alkaloids of traditional Chinese medicine Yanhuanglian in rats following intravenous and oral administration. J. Pharm. Biomed. Anal..

[22] Gao Y., Hu S., Zhang M., Li L.L., Lin Y.N. (2014). Simultaneous determination of four alkaloids in mice plasma and brain by LC-MS/MS for pharmacokinetic studies after administration of Corydalis Rhizoma and Yuanhu Zhitong extracts. J. Pharm. Biomed. Anal..

[23] Pan G.Y., Wang G.J., Liu X.D., Fawcett J.P., Xie Y.Y. (2002). The Involvement of P-glycoprotein in berberine absorption. Pharmacol. Toxicol..

[24] Maeng H.J., Yoo H.J., Kim I.W., Song I.S., Chung S.J., Shim C.K. (2002). P-glycoprotein-mediated transport of berberine across Caco-2 cell monolayers. J. Pharm. Sci..

[25] Wu X., Li Q., Xin H., Yu A., Zhong M. (2005). Effects of berberine on the blood concentration of cyclosporin A in renal transplanted recipients: clinical and pharmacokinetic study. Eur. J. Clin. Pharmacol..

[26] Liang M., Zhang W., Hu J., Liu R.H., Zhang C. (2006). Simultaneous analysis of alkaloids from Zanthoxylum nitidum by high performance liquid chromatography-diode array detector-electrospray tandem mass spectrometry. J. Pharm. Biomed. Anal..

[27] Cai M. (2007). Studies on the Chemical Constituents and Analysis Methods for Several Traditional Chinese Medicines. Ph.D. Thesis.

[28] Jia C.P., Huang X.L., Li Y., Feng F. (2013). Analysis of alkaloids in Zanthoxylum nitidum by HPLC-DAD/ESI-Q-TOF-MS. Chin. J. Chin. Mater. Med..

[29] Matuszewski B.K., Constanzer M.L., Chavez-Eng C.M. (2003). Strategies for the assessment of matrix effect in quantitative bioanalytical methods based on HPLC-MS/MS. Anal. Chem..

